# Abdominal Bloating and Distension: Common Symptoms but Limited Evidence

**DOI:** 10.1002/ueg2.70108

**Published:** 2025-09-06

**Authors:** Juha Taavela, Mohamed G. Shiha

**Affiliations:** ^1^ Department of Gastroenterology and Alimentary Tract Surgery Tampere University Hospital Tampere Finland; ^2^ Celiac Disease Research Center Faculty of Medicine and Health Technology Tampere University Tampere Finland; ^3^ Division of Clinical Medicine School of Medicine and Population Health University of Sheffield Sheffield UK; ^4^ Department of Gastroenterology University Hospitals of Leicester Leicester UK

That infuriating feeling that everyone has endured at some point in life, usually surpassing but not frequently recurring, is abdominal bloating and distension. But what happens when the sensations stay with you all the time and disrupt your daily life? Such patients are common in the general population, with global prevalence rates reaching up to 20% [1], and they present a challenge for the treating physician. Patients with functional bloating and distension experience a decreased quality of life and an increased frequency of doctor visits [2]. Such patients need information on the causes of their condition and how to manage it. These common issues are now addressed in a new consensus document by Melchior et al., published in this issue of *United European Gastroenterology Journal* [3]. The recommendations provide both physicians and patients with a clear diagnostic flowchart and management portfolio with available evidence, aiming to ensure the best quality of care for both parties.

When evaluating patients with bloating and distension, it is of utmost importance to consider differential diagnoses appropriately: *Is it functional, or should other causes be excluded?* The authors of the consensus provide a simple, much‐needed flowchart on this issue. Basic tests—such as celiac disease antibodies, thyreotropin, and glucose/HbA1c—along with the exclusion of alarm signs, are always mandatory to rule out organic pathology. Importantly, the consensus supports recent European recommendations against the use of breath tests to diagnose small intestinal bacterial overgrowth (SIBO) [4], ending a longstanding but problematic practice that often led to injudicious use of antibiotics and patient harm without supporting evidence. Whether additional investigations are needed depends on the physician's physical examination, the detailed patient history, and whether the Rome IV criteria for functional bloating and distension are met [3].

Evidence for the management of functional abdominal bloating and distension remains limited [3]. Most of the treatment recommendations in the current consensus are extrapolated from randomized controlled trials conducted in patients with irritable bowel syndrome (IBS) who experience bloating and distension. However, in many of these trials, improvement in bloating and distension symptoms was not the primary endpoint, and the study populations differ from patients with functional abdominal bloating and distension who may have infrequent abdominal pain or no change in bowel habit and therefore do not meet the Rome criteria for IBS. Subsequently, the applicability of these findings to the broader population with isolated functional bloating and distension is uncertain and therapeutic decisions should be made with careful consideration of these limitations. In clinics, physicians should remain aware of this uncertainty and discuss it openly with patients when considering management options.

The current consensus recommendations highlight the substantial evidence gap surrounding functional abdominal bloating and distension. Despite being a highly prevalent and burdensome condition, most of the statements within the consensus were not suitable for formal quality grading due to the lack of specific high‐quality data. The value of the included good practice statements may be questioned, as they can at times appear self‐evident. Nonetheless, the consensus offers a valuable framework to guide clinical decision‐making by providing evidence‐based recommendations where available and clearly acknowledging areas of uncertainty, thereby often supporting shared decision‐making between clinicians and patients.

## Conflicts of Interest

J.T. and M.S. are Trainee Editors in United European Gastroenterology Journal.

## Data Availability

The authors have nothing to report.
